# Levamisole-Induced ANCA Vasculitis and Cutaneous Necrosis

**Published:** 2014-10-17

**Authors:** Matthew Tichauer, Shawn Fagan, Jeremy Goverman

**Affiliations:** ^a^Department of Surgery, Acute Care Surgery, and Surgical Critical Care; ^b^Division of Trauma, Acute Care Surgery, and Surgical Critical Care; ^c^Division of Burn Surgery, Massachusetts General Hospital, Boston, Mass

**Keywords:** Levamisole, ANCA, Vasculitis, skin necrosis, cutaneous necrosis

**Figure:**
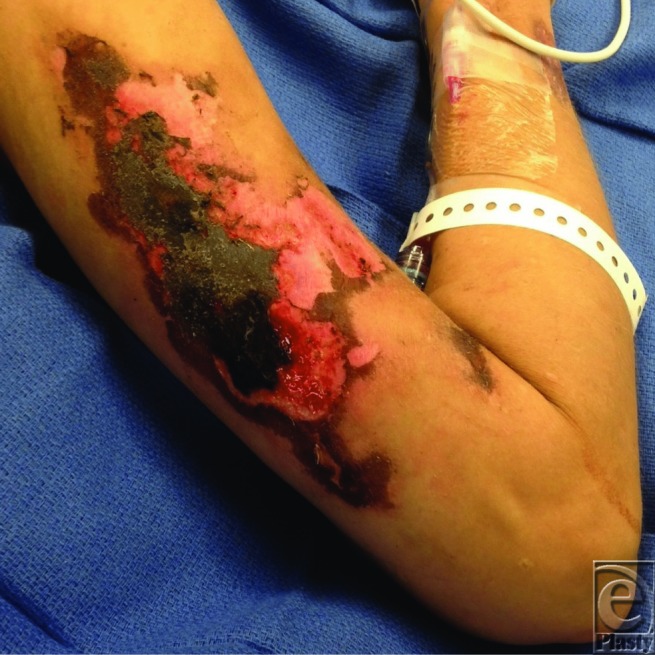


**Figure:**
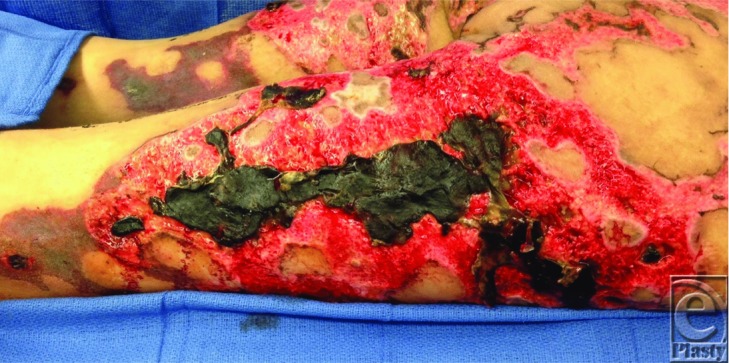


## DESCRIPTION

A 34-year-old woman with a history of intravenous drug use (cocaine/heroin) presented with cutaneous necrosis of the bilateral anterior/posterior thighs, buttocks, and left upper extremity, determined to be a complication of levamisole-induced antineutrophil cytoplasmic antibody (ANCA) vasculitis.

## QUESTIONS

1. **How is ANCA-associated vasculitis diagnosed?**
2. **What is the pathophysiology behind ANCA-associated vasculitis?**
3. **What are the clinical features of ANCA-associated vasculitis?**
4. **What are the histological findings in drug-induced ANCA-associated vasculitis?**

## DISCUSSION

Immunoflourescence is a screening tool utilized to detect for the presence of ANCAs. Antibiodies to specific antigens are then detected by enzyme-linked immunosorbent assay. Thus the order of diagnostics is a positive immunoflourescence test followed by enzyme-linked immunosorbent assay to specify the antibodies present.^1^ In drug-induced ANCA-associated vasculitis, combined positivity of both anti-myeloperoxidase and antiproteinase 3 (PR3) antibodies is occasionally seen, however, rarely in idiopathic forms of the disease.^2^

ANCA activate neutrophils and monocytes that express the ANCA antigens, proteinase 3, and myeloperoxidase, on their surface. The neutrophils are then activated and they adhere to the endothelial cells, resulting in the release of proteolytic granules and proinflammatory cytokines. Inflammatory resolution is halted by ANCAs disrupting neutrophil apoptosis, preventing removal of dead cells, and resulting necrosis.^3^

Patients presenting with ANCA-associated vasculitis typically present with constitutional symptoms such as myalgias, fever, anorexia, malaise, weight loss, and diaphoresis. ANCA-associate vasculitis can affect all organ systems. Involvement of the upper and lower respiratory tracts and the kidneys manifest with symptoms such as cough, hemoptysis, rhinorrhea, epistaxis, sinusitis, otitis media, hematuria, proteinuria, and the presence of red cell casts. Progression to severe lung disease is common as is deterioration in renal function. With ocular involvement, patients may present with episcleritis, uveitis, proptosis, and optic nerve ischemia. Ischemia and hemorrhage from the gut as well as myocardial ischemia may occur as a cardiac manifestation.^3^ Agranulocytosis has also been reported in patients with cocaine/levamisole abuse.

Patients with drug-induced ANCA-associated cutaneous necrosis present with heterogeneous cutaneous manifestations including necrotic lesions, purpura, abscesses, bullous skin lesions, extensive ear, and digital involvement.^4^

The histologic findings of the cutaneous lesions associated with levamisole-induced ANCA vasculitis show thrombotic vasculitis or leukocytoclastic vasculitis, at times in the presence of vascular occlusions.^5^^,^^6^
